# The Impact of Novel Coronavirus on Dermatology

**DOI:** 10.5826/dpc.1002a49

**Published:** 2020-03-31

**Authors:** Vincenzo Piccolo, Giuseppe Argenziano

**Affiliations:** 1 Dermatology Unit, University of Campania, Naples, Italy

The outbreak of the novel coronavirus infection (COVID-19) in China in December 2019 and the rapid subsequent spread of the infection worldwide have created awareness among patients and doctors. To date (March 20, 2020) 250,856 confirmed cases and 10,389 deaths have been reported [1]. Beyond the important health issue, effects on economy, education, social life, and tourism have arisen.

Most hospitals in the affected countries are full of real and suspected cases, therefore overloading health professionals as has rarely happened before. The emergency mostly involves internal medicine departments, including infectious diseases, respiratory medicine, and emergency and intensive care units. To a lesser extent, other specialties have been hit by the problem as well. What happened to dermatology?

In a couple of weeks we have thoroughly changed our way of doing things, and frightened patients have contributed to these changes, producing some effects that are without precedent:

1. Consultations in outpatient clinics have considerably declined in number for two main reasons: patients are afraid and dermatologists recommend avoiding non-urgent visits.
2. Governments (as happened in Italy, for instance) have stopped all visits to outpatient clinics except for oncology.
3. Dermatological consultation requires close contact with patients, thus producing fear among doctors of being infected, consequently reducing the quality of performance.
4. Although authorities recommend the use of protective masks for health practitioners, most of them have not been provided because of the high demand of these disposables.
5. Surgery for skin cancer is often postponed according to the patient’s will.
6. Patients stop taking medications independently, in particular, immunosuppressants and biological drugs, thinking their use leads to a higher risk of developing coronavirus infection.

The list of consequences for our practice could be endless, and the same is likely in other medical specialties as well. The virus, along with the fear of the virus, has undoubtedly changed our lives. It is not easy to establish what the right thing to do is and, although isolation is mandatory to control the infection, postponing some medical procedures could be lethal to some patients.

Specialty-specific recommendations should be urgently available for each branch of medicine to avoid self-management and confusion among clinicians and subsequent serious consequences to patients.
